# Incorporating Technology Into the iCook 4-H Program, a Cooking Intervention for Adults and Children: Randomized Controlled Trial

**DOI:** 10.2196/11235

**Published:** 2019-08-29

**Authors:** Sarah Colby, Lauren Moret, Melissa D Olfert, Kendra Kattelmann, Lisa Franzen-Castle, Kristin Riggsbee, Magen Payne, Ainsley Ellington, Cary Springer, Chelsea Allison, Sa'Nealdra Wiggins, Rochelle Butler, Douglas Mathews, Adrienne A White

**Affiliations:** 1 Department of Nutrition University of Tennessee Knoxville, TN United States; 2 Davis College of Agriculture, Natural Resources, and Design West Virginia University Morgantown, WV United States; 3 Health and Nutritional Sciences Department South Dakota State University Brookings, SD United States; 4 College of Education and Human Sciences University of Nebraska-Lincoln Lincoln, NE United States; 5 Research Computing Support University of Tennessee Knoxville, TN United States; 6 University of New England Portland, ME United States; 7 School of Food and Agriculture University of Maine Orono, ME United States

**Keywords:** technology, videos, intervention, cooking, child health

## Abstract

**Background:**

Families who cook, eat, and play together have been found to have more positive health outcomes. Interventions are needed that effectively increase these health-related behaviors. Technology is often incorporated in health-related interventions but is not always independently assessed.

**Objective:**

The objective of this study was to describe challenges and facilitators to incorporating technology into the iCook 4-H intervention program.

**Methods:**

Dyads (n=228) composed of children (mean 9.4, SD 0.7 years old) and an adult primary meal preparer (mean 39.0, SD 8 years) were randomly assigned to a control (n=77) or treatment group (n=151). All treatment group dyads participated in 6 in-person sessions designed to increase families cooking, eating, and playing together. We incorporated Web-based between-session technological components related to the curriculum content throughout the intervention. Assessments were completed by both groups at baseline and at 4, 12, and 24 months; they included measured anthropometrics for children, and online surveys about camera and website skill and use for dyads. Session leaders and participants completed open-ended process evaluations after each session about technological components. We computed chi-square analysis for sex differences in technological variables. We tested relationships between video posting frequency and outcomes of interest (cooking frequency, self-efficacy, and skills; dietary intake; and body mass index) with Spearman correlations. Process evaluations and open-ended survey responses were thematically analyzed for beneficial and inhibiting factors, including technological components in the curriculum.

**Results:**

Only 78.6% (81/103) of children and 68.3% (71/104) of adults reported always being comfortable accessing the internet postintervention. Boys reported being more comfortable than girls with technological tasks (*P*<.05). Children who posted more videos had a higher level of cooking skills at 4 months postintervention (*r*=.189, *P*=.05). Barriers to website usage reported most frequently by children were lack of accessibility, remembering, interactivity, motivation, time, and lack of parental encouragement.

**Conclusions:**

Incorporating technological supports, such as cameras and websites, into children’s programs may help produce improved outcomes. Identifying barriers to and patterns of technology usage need to be considered when developing future child health promotion interventions.

**Trial Registration:**

ISRCTN Registry ISRCTN54135351; https://www.isrctn.com/ISRCTN54135351

## Introduction

### Background

Unhealthy dietary patterns in childhood are associated with less than optimal growth patterns, cognitive deficiencies, emotional unwellness, and the development of many chronic diseases [1-7]. This is of concern because few children in the United States meet all dietary intake recommendations [1]. Healthier dietary behavior established in childhood has been associated with decreased lifelong risk of many chronic diseases, including obesity, diabetes, cardiovascular disease, and some cancers [8-11]. With current dietary patterns potentially increasing the risk for chronic disease later in life, it is important to develop effective intervention strategies to improve dietary behaviors among children.

While there is varying success with interventions designed to improve diet patterns of children, some technology-based interventions have been found to be more effective than nontechnology-based interventions [12-14]. Researchers have found that interventions designed with both face-to-face and technological strategies are more effective than similar interventions with only face-to-face components [15]. Technological strategies and supports can include a wide range of approaches.

Social media technology is a tool that can be used in health promotion interventions for children because children are often one of the earliest adopters of technology [16-18]. Although there is limited research on health-related interventions using social media, it is an increasingly used strategy and more research is needed to determine its effectiveness and influence on programmatic outcomes [19,20]. Many successful social media sites (eg, Facebook, Snapchat, Twitter, Instagram, and YouTube) have user-generated content (UGC) as the primary source of content [21]. The use of online UGC in interventions designed for children, including user-created videos posted to a website, may have the potential to increase program engagement leading to success. However, it is a largely underresearched intervention strategy.

### Objective

The purpose of this study was to describe the incorporation of technology, including uses of and barriers to the use of technology, during an intervention by program participants; specifically, this study examined the use of technology in a child-adult dyad intervention program focused on cooking, eating, and playing together, called iCook 4-H.

## Methods

### Setting and Participants

The iCook 4-H intervention program was a pre-post, follow-up intervention study conducted over 2 years for dyads (9- to 10-year-old children and their primary adult meal preparer) across 5 states in the United States (Maine, Nebraska, South Dakota, Tennessee, and West Virginia). Of those adults who reported their relationship to the children (160/228, 70.2%), 151 (94.4%) of the primary adult meal preparers were parents, 6 (3.8%) were grandparents, and 3 (1.9%) were another adult.

The purpose of iCook 4-H, a series of 6 cooking lessons, was to help families learn to cook, play, and eat together to assist in the prevention of childhood obesity. After a year of curriculum development and pilot testing, we recruited 228 child-adult dyads in August 2013 using flyers, newspaper and radio advertisements, posters, emails, and postings on social media. Participants recruited for this study (1) were free from life-threatening illness or conditions, (2) were free from food allergies or activity-related medical restrictions that would prevent participation in a face-to-face nutrition and fitness program, (3) were willing to eat meat and dairy foods, and (4) had regular access to a computer with an internet connection. Participant recruitment efforts targeted low-income, rural, and diverse populations by distributing recruitment materials in communities in the 5 intervention sites.

Although this study was not prospectively registered as a randomized controlled trial, the institutional review boards at all participating universities approved the study procedures. All participants assented and consented to participate. The trial is reported in accordance with the Consolidated Standards of Reporting Trials of Electronic and Mobile Health Applications and Online Telehealth (CONSORT-EHEALTH) checklist (Multimedia Appendix 1 [22]).

### Study Design

We randomly assigned those who met the inclusion criteria to the control group (n=77) or intervention group (n=151), using a pattern of 1 control for every 2 treatment dyads. All participants completed baseline (0-month), postintervention (4-month), and follow-up (12- and 24-month) assessments. Assessments at these time points included measuring children’s height, weight, waist circumference, and blood pressure as well as completing surveys. Survey questions assessed demographics, dietary intake, food security, cooking frequency, and cooking self-efficacy; program evaluation questions focused on cooking skills, family meals, physical activity, and goal setting [23-31]. We added questions after 12 months of the project to assess engagement with technological self-efficacy (ie, accessing the internet, and creating and uploading digital photos and videos to a study website). At 24 months, we added open-ended questions about website usage, barriers to technology use, and preferences for technology. Stipends of US $80 were provided to dyads, evenly distributed among the 4 assessment periods for those who completed each one. Control group participants had no other interactions with the researchers beyond the assessments during the 24-month study. The purpose of the control group was to provide a group to compare with the intervention group during analysis; we conducted this analysis to determine the effectiveness of the larger iCook randomized controlled trial in preventing excessive weight gain among children through increased family cooking, mealtime, and physical activity.

Treatment dyads participated in six 2-hour sessions held every other week, over a period of 12 weeks. Session leaders were Extension personnel or graduate students in nutrition- and health-related fields. The Extension personnel were community nutrition educators or paraprofessionals from the participating land-grant institutions. At the end of each session, leaders and dyads completed online process surveys, which included open-ended feedback questions on technology. Leaders also participated in monthly phone calls with researchers for process evaluation. Each 2-hour session included dyad-centered focus areas on culinary skills, food preparation, physical activity, family mealtime and communication, and goal setting.

We developed a password-protected website for participants to use to reinforce session content and increase connections between participants across the 5 states through status updates and comments. Participants were asked to post videos, recipes, status updates about personal goals, and reactions to other participants’ postings between sessions. Videos were to be 3 to 5 minutes in length and reflect topics learned in the sessions. Video cameras were provided to the treatment group, and technological training on cameras and the study website was provided at session 1.

Beginning 1 month after the 12-week program concluded and continuing for 18 months, participants received an additional 21 months of website activities, monthly newsletters, and quarterly in-person booster sessions. For the website activities, we asked dyads to continue to create and post cooking, family mealtime, and physical activity videos. Weekly physical activities and monthly food challenges were also posted on the website [32]. An example physical activity challenge was “the plank challenge,” which was a balancing *pose* to strengthen arms and spine while toning abdominal muscles. The challenge was to hold the plank pose longer each day over the week of the challenge [33,34]. An example of a monthly food challenge was “the purple food challenge,” where participants were asked to cook with a new purple food. Status updates posted by children about their physical and food challenges were entered into monthly drawings to receive monetary awards ranging in value from US $10 to $50, depending on the type and length of the challenge. In addition to having access to the website, children also received an age-appropriate newsletter through the mail. The newsletters contained the same content that we posted on the website. Quarterly booster sessions encouraged participants to continue the study goals of cooking, eating, and playing together. Activities at the booster sessions included bowling, field days, and picnics for sharing new recipes.

### Development of Themes

To determine the issues related to website usage, 1 researcher (SC) reviewed the participants’ and leaders’ open-ended process survey responses to develop a codebook for thematic analysis [35,36]. Then 2 researchers (SW, CA) independently coded the open-ended survey responses using the provided codebook. Finally, a third researcher (SC) compared the codes and resolved any discrepancies that existed between the coders. We then collapsed the codes into larger groups of findings that became the themes [35,36]. We used these themes to develop an understanding of facilitators and barriers related to the iCook website.

### Statistical Analysis

We calculated frequency statistics for demographics, technological variables, and website usage and preferences for children and adults in the treatment group. We grouped participants by video posting frequency (none: 0 videos; low: 1-3 videos; moderate: 4-7 videos; and high: ≥8 videos). Chi-square analysis determined whether differences existed by sex for technological variables, website usage and preferences, and video posting frequency. Spearman correlations investigated relationships between video posting frequency and outcomes of interest (cooking skills, dietary intake [fruit, vegetable, whole grain, dairy, and saturated fat], and body mass index [BMI]) at 4 months.

## Results

### Participant Characteristics

**Figure 1:**
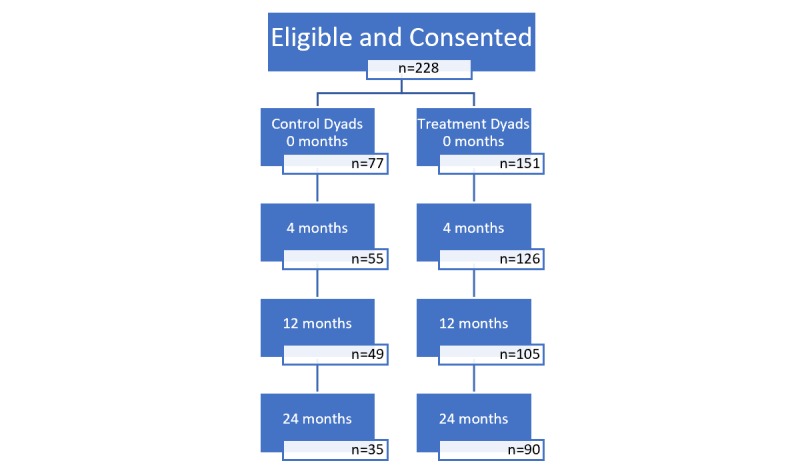
iCook intervention study participation flow diagram.

Dyads (n=228) consisted of a child (mean age 9.4, SD 0.7 years) and an adult primary meal preparer (mean age 39.0, SD 8 years). Figure 1 shows the flow of the control and treatment groups through the study.

Most child participants were white (135/201, 67.2%), with over half being female (114/208, 54.8%). A total of 33.0% (66/201) of participating households were food insecure (Table 1), and 26.0% (54/208) of adults reported participating in at least one food assistance program.

**Table 1:** Adult participant demographic information at baseline for control and treatment groups in the iCook 4-H intervention program (n=209).

Characteristics		Value, n (%)
**Sex (n=209)**		
	Female	188 (90.0)
	Male	21 (10.0)
**Ethnicity/race (n=201)**		
	White	155 (77.1)
	Black	16 (8.0)
	Hispanic	13 (7.0)
	Other	17 (9.0)
**Marital status (n=208)**		
	Married	145 (69.7)
	Not married	63 (30.3)
**Educational level attained (n=225)**		
	Less than high school	12 (5.3)
	High school	27 (12.0)
	Associate degree	28 (12.4)
	Some college or university	59 (26.2)
	Bachelor’s degree	66 (29.3)
	Graduate school	33 (15.0)
**Household food security (n=201)**		
	Food insecure	66 (33.0)
	Food secure	135 (67.2)

### Survey Results

At 12 months, 100.0% (103/103) of the children reported having access to the internet, with 83.4% (86/103) accessing the internet through a personal computer, 15.0% (15/103) using mobile devices, 1.0% (1/103) accessing the internet through work or school, and 1.0% (1/103) using a gaming console; 78.6% (81/103) of children and 68.3% (71/104) of adults in the treatment group reported that they were always comfortable accessing the internet (Table 2). The only differences identified in the chi-square analyses were with sex of the child and the following activities: accessing the internet (χ^2^_4_=10.2, *P*=.04), downloading videos onto a computer (χ^2^_4_=10.9, *P*=.03), and putting videos online (χ^2^_4_=12.5, *P*=.01). More boys than girls reported being very comfortable with accessing the internet (45/93, 48.4% vs 58/112, 52.0%, respectively), downloading videos (35.8%, 33/93 vs 16/112, 14.0%, respectively), and putting videos online (32/93, 34.6% vs 14/112, 12.2%).

Although all treatment group children were asked to access the website and submit postings of their videos, only 69.0% (71/103) went on the iCook website. Of those who did post videos, 59% (42/71) posted 1 to 3 videos, 24% (17/71) posted 4 to 7 videos, and 17% (12/71) posted 8 or more videos. One person posted 26 videos and 1 person posted 29 videos. The most commonly reported reason why children visited the website was the videos, followed by functionality, recipes, information, challenges, cooking ideas, and activities. The top barriers to using the website that children reported were accessibility issues, forgetfulness, lack of interactivity, motivation, time, and lack of parental encouragement. The top barriers for children using the website reported by adults paralleled the children’s reports: forgetfulness, accessibility, lack of interactive games, and time, along with parental restriction on the child’s computer time. Of the 53 treatment group adult participants who completed the 24-month postintervention surveys and reported receiving the newsletters, 37 (70%) preferred the newsletter over the website. Reasons for preferring the newsletter included that receiving the physical newsletter provided a reminder to look at the content and that it was easy to take with the family out of the house.

Session leaders identified 4 main technological issues, barriers, and limitations in the process evaluations: (1) changing participant preference for recording device from cameras to cell phones; (2) access to adequate upload speeds, which were disproportionally slower for lower-income families; (3) lack of technological knowledge and skills for children, adults, and session leaders; and (4) difficulties creating motivation and habit to use the program website.

At 4 months, children who posted more videos also reported a higher level of cooking skills (*r*=.189, *P*=.05). Frequency of posting had no relationship with any other outcomes of interest (children’s dietary behaviors, cooking self-efficacy, family togetherness, or BMI).

**Table 2:** Treatment participants’ self-efficacy for technological skills related to the iCook 4-H intervention program.

Survey item (“I can...”)		Response regarding level of comfort				
		Never, n (%)	Rarely, n (%)	Sometimes, n (%)	Most of the time, n (%)	Always, n (%)
**Access the internet**						
	Children (n=103)	1 (1.0)	2 (1.9)	12 (11.7)	7 (6.8)	81 (78.6)
	Adults (n=104)	3 (2.9)	2 (1.9)	9 (8.7)	19 (18.3)	71 (68.3)
**Take digital pictures**						
	Children (n=102)	4 (3.9)	3 (2.9)	5 (4.9)	12 (11.8)	78 (76.5)
	Adults (n=104)	3 (2.9)	1 (1.0)	8 (7.7)	20 (19.2)	72 (69.2)
**Download digital pictures onto a computer**						
	Children (n=102)	29 (28.4)	17 (16.7)	21 (20.6)	9 (8.8)	26 (25.5)
	Adults (n=104)	6 (5.8)	10 (9.6)	19 (18.3)	21 (20.2)	48 (46.2)
**Record digital videos**						
	Children (n=101)	7 (6.9)	5 (5.0)	14 (13.9)	10 (9.9%)	65 (64.4)
	Adults (n=103)	3 (2.9)	10 (9.7)	12 (11.7)	30 (29.1)	48 (46.6)
**Download videos onto a computer**						
	Children (n=103)	32 (31.1)	16 (15.5)	18 (17.5)	11 (10.7)	26 (25.2)
	Adults (n=101)	12 (11.9)	19 (18.8)	16 (15.8)	25 (24.8)	29 (28.7)
**Upload videos to a website**						
	Children (n=101)	39 (38.6)	13 (12.9)	18 (17.8)	7 (6.9)	24 (23.8)
	Adults (n=102)	20 (19.6)	252 (24.5)	21 (20.6)	16 (15.7)	20 (19.6)

## Discussion

### Principal Findings

Although increased posting of videos was not related to changes in children’s dietary behaviors, cooking self-efficacy, family togetherness, or BMI, it was associated with increased cooking skills. It is possible that posting of videos was only an indication of how engaged the children were overall in the program and not causally associated with improving cooking skills. It is also possible that as children cooked more while making videos (and experienced repeated exposure to the cooking concepts while reviewing videos), they increased their cooking skills. Future experimental research is needed to assess the impact of making cooking videos on children’s cooking skills to determine causality.

The main purpose of this study was to describe the uses of and barriers to technology in the iCook 4-H intervention program. We did not specifically test the impact of students creating UGC in this study, since there was not a group that received the face-to-face intervention without the incorporation of technology. Most online UGC videos related to learning have been developed and tested for college and university populations [37,38]. Although investigations of the effectiveness of children creating videos to increase the effectiveness of class-based learning experiences are largely lacking, children creating their own UGC videos might be expected to create more excitement and engagement in program activities in part because children have an affinity for technology and online activities [39]. When children create UGC videos based on information from an in-person class, they are required to reflect on the content, synthesize information, and reinforce learning through repetition. The exercise of reflection provides children with a clear connection between the new material and previous knowledge [40]. Synthesizing requires a deeper understanding of the learned information to successfully translate and communicate material [41]. Repetition and reintroducing content is important in the learning process [42]; not only did children in our study repeat and practice skills as they made videos, but they were also repeatedly exposed to the information when they shared the videos they created and watched them with friends and family. When children created videos they also became ambassadors of the message, thereby increasing the likelihood that they would adopt the behavior because of social desirability to be in congruence with what they were saying to others; if they “walked the walk” then they were more likely to “talk the talk” [43,44]. When they were physically creating the videos they were using kinesthetic and active experiential learning techniques, which have also been found to improve learning outcomes [45]. If future research is developed to investigate the impact of this video creation strategy, many aspects related to technology need to be considered.

Limited technological self-efficacy of participants in this study needs to be considered. Even after technological training and participation in a 6-session program that included technology as a continuous component, many participants were not comfortable with basic technological skills (such as accessing the internet). Many researchers developing interventions may be immersed in a world in which technology has saturated most aspects of daily life. These researchers may not be aware of the technological disparities that may exist in less-affluent communities. Data indicate that most individuals in the United States, despite economic status, have access to the internet, but this may not accurately reflect technological disparities in self-efficacy and skills [46,47]. Although all participants in this study did have access to the internet, this may not accurately reflect the proportion of individuals who have access in these communities. Access to the internet was an advertised requirement for participation in this project. Thus, the actual access to skills and self-efficacy with technology in these communities may be overrepresented in this sample. This concept of technological disparities may be similar to health disparities and health literacy disparities, and deserves further investigation.

Additionally, specific to this study, participants were asked to create videos showing themselves at home cooking, eating, and being active with their families. Participants were advised to keep videos to a short length of time (3-5 minutes). Because cooking takes place over a longer period than was recommended for the video length, participants needed to be able to edit cooking videos. The ability to edit a video is an advanced technological skill. With 15.0% (15/103) of the participants accessing the internet through mobile devices, this may have added an additional barrier to participation that needs to be considered. As mobile devices become easier to use and more on par with other computing technologies, this may become less of a barrier.

The ability to test experimentally the effectiveness of this technological approach in the future would likely be limited by technological skills. After technological training and participation in this program, many participants were still not comfortable with skills needed to effectively participate in the technological aspects of the program (creating and uploading videos to a website). Interestingly, children were a little more comfortable than adults in making videos and adults were a little more comfortable than children in downloading the videos to a computer and uploading them to a website. This may reflect roles the participants self-selected to complete during their participation in the project. It is also worth noting that both children and adults were more comfortable in taking and uploading digital photographs to a website than working with videos. These differing levels of technological skills should be considered when developing future research programs.

Although limited technological skills were not identified by participants as a reason for preferring the newsletter over the website, it is interesting that participants preferred printed materials over Web-based material. However, caution is needed when interpreting the preferences reported for the newsletters over website material found in this study. It is possible that what we observed was not because of differences in preferred communication strategies but instead was specific to materials developed for this study. Researchers have found that, although online technologies are beginning to be used in interventions, many websites are lacking components necessary to be effectively used [48]. Another possible explanation for the preference for print materials is that, if participants were saturated with information from a variety of other electronic formats in other aspects of their life, receiving a printed newsletter in the mail might have been a novelty. It is also possible that participants might have had negative experiences with technology when trying to create and upload videos, and those frustrations affected their overall feelings toward use of the website. We did not anticipate these strong preferences for printed material. Other researchers have found that 90% of parents surveyed wanted Web-based interventions to help manage childhood obesity [49]. More research is needed to understand the communication preferences observed in this study. Research related to technology also needs to be continuously and frequently reinvestigated because type, access, familiarity, comfort, and skills related to technology change rapidly. The participants in this study may have very different experiences, skills, and preferences for technology even a few years later.

Many lessons learned about issues related to the incorporation of technology and UGC in this child health promotion intervention may be valuable to other researchers as they design future interventions. When this project was originally planned, mobile phones were less ubiquitous and cameras were provided to participants so that they could make their videos. By the end of the project, more participants had and preferred using their own smartphone-style mobile phones over other camera recording equipment. Future programs incorporating UGC videos may not need to incorporate the cost of providing cameras to participants (even when working with low-income communities) [47,50].

Although many participants had internet access at home and download speeds were not a reported barrier, we found that upload speeds varied. Limited upload speeds were a barrier to uploading videos for many families. Without adequate upload speeds, the time required to upload videos was impractical for many participants. We anticipate that with technological advances, access to sufficient upload speeds will become more widespread; however, the timeline for that progression is unknown and this barrier to uploading UGC (specifically videos) needs to be considered when developing interventions that incorporate these technological strategies. This is an especially important consideration when working with low-income communities that may not be able to afford more expensive internet services that have faster upload speeds [47].

Some of the community sites where sessions were taught also had limited or no access to the internet. This barrier made the incorporated technological instruction challenging and prevented the participants from being able to have lesson leaders assist with video uploading before or after the in-person sessions. Mobile hotspots were used to overcome this issue for technological training in some locations but were not adequate to overcome the barrier of upload time requirements. Although with expanding internet access, it is likely that this will be less of a problem in future interventions, internet access is an area that needs to be considered when developing technology-based community interventions.

When the project began, there were no widely used existing social media platforms that allowed children and parents to interact in password-protected or closed online environments; thus, a password-protected website was created for use in this study. The password-protected site was needed to increase safety for the children and to alleviate concerns that parents and session leaders voiced about children interacting in online environments. However, encouraging participants to visit a newly created website and use it on an ongoing and frequent basis was challenging. Also, since videos needed to be uploaded as private YouTube files and the link then transferred to the iCook 4-H site, there may have been too many steps for participants to deal with to complete the process. A website community that would be self-generating did not arise, probably due to the relatively small number of participants available for website interaction. If a mobile app were available, it may help to increase child and adult participation in creating and uploading videos.

By the end of the study, options for creating closed communities were available on many popular social media platforms. Some researchers have had success initiating observations of successful, naturally occurring social media communities established for specific health conditions [51,52]. However, other researchers have reported limited success in their efforts to start and maintain communication about health topics on similar sites [53]. Despite conflicting research, it may be beneficial to avoid creating new websites for future interventions due to the financial and time costs needed to develop and maintain the site. Instead, future interventions using UGC online may benefit from incorporating their program into existing, familiar, and high-traffic sites; many of these commonly used sites now have the option to have closed or private, child-specific, or moderated groups.

### Limitations

Although this study contributed novel perspectives in an emerging area of research, there were limitations in the study design. We did not test the effectiveness of incorporating UGC into the study design. Most participants did not participate or participated minimally in creating UGC. Because of the small sample size, this was not a representative sample of a larger national audience and the results cannot be generalized.

### Conclusions

Overall, we have provided valuable perspectives on use and barrier issues that may be encountered when incorporating technology and UGC videos into programs designed to promote health for children. The effect of specifically incorporating UGC in child health promotion interventions needs to be tested with a randomized controlled trial design to isolate and test the impact of the children creating content on behavior outcomes. In this future research, the preferred communication strategies of the target population and barriers to participation in the technological components of the program need to be assessed and addressed prior to intervention implementation. This would allow for the development and implementation of an intervention that would have adequate and consistent levels of participation in the development of UGC material. This future research is needed to establish the impact of children creating videos on health-related behavior.

## Appendix

Multimedia Appendix 1 CONSORT-EHEALTH checklist (V 1.6.1).

## Abbreviations

BMI: body mass index
CONSORT-EHEALTH: Consolidated Standards of Reporting Trials of Electronic and Mobile Health Applications and Online Telehealth
UGC: user-generated content

## References

[1] Banfield EC, Liu Y, Davis JS, Chang S, Frazier-Wood AC (2016). Poor adherence to US dietary guidelines for children and adolescents in the National Health and Nutrition Examination Survey population. J Acad Nutr Diet.

[2] Marshall S, Burrows T, Collins CE (2014). Systematic review of diet quality indices and their associations with health-related outcomes in children and adolescents. J Hum Nutr Diet.

[3] Haapala EA, Eloranta A, Venäläinen T, Schwab U, Lindi V, Lakka TA (2015). Associations of diet quality with cognition in children - the Physical Activity and Nutrition in Children Study. Br J Nutr.

[4] Kulkarni AA, Swinburn BA, Utter J (2015). Associations between diet quality and mental health in socially disadvantaged New Zealand adolescents. Eur J Clin Nutr.

[5] McMartin SE, Willows ND, Colman I, Ohinmaa A, Storey K, Veugelers PJ (2013). Diet quality and feelings of worry, sadness or unhappiness in Canadian children. Can J Public Health.

[6] Perry CP, Keane E, Layte R, Fitzgerald AP, Perry IJ, Harrington JM (2015). The use of a dietary quality score as a predictor of childhood overweight and obesity. BMC Public Health.

[7] Ogata BN, Hayes D (2014). Position of the Academy of Nutrition and Dietetics: nutrition guidance for healthy children ages 2 to 11 years. J Acad Nutr Diet.

[8] Kwon S, Janz KF, Letuchy EM, Burns TL, Levy SM (2015). Active lifestyle in childhood and adolescence prevents obesity development in young adulthood. Obesity (Silver Spring).

[9] Günther ALB, Schulze MB, Kroke A, Diethelm K, Joslowski G, Krupp D, Wudy S, Buyken AE (2015). Early diet and later cancer risk: prospective associations of dietary patterns during critical periods of childhood with the GH-IGF axis, insulin resistance and body fatness in younger adulthood. Nutr Cancer.

[10] Kaikkonen JE, Mikkilä V, Magnussen CG, Juonala M, Viikari JSA, Raitakari OT (2013). Does childhood nutrition influence adult cardiovascular disease risk?--insights from the Young Finns Study. Ann Med.

[11] Dorgan JF, Liu L, Barton BA, Deshmukh S, Snetselaar LG, Van Horn L, Stevens VJ, Robson AM, Lasser NL, Himes JH, Shepherd JA, Pourfarzib R, Pettee Gabriel K, Kriska A, Kwiterovich PO (2011). Adolescent diet and metabolic syndrome in young women: results of the Dietary Intervention Study in Children (DISC) follow-up study. J Clin Endocrinol Metab.

[12] Ho M, Garnett SP, Baur L, Burrows T, Stewart L, Neve M, Collins C (2012). Effectiveness of lifestyle interventions in child obesity: systematic review with meta-analysis. Pediatrics.

[13] Colquitt JL, Loveman E, O'Malley C, Azevedo LB, Mead E, Al-Khudairy L, Ells LJ, Metzendorf M, Rees K (2016). Diet, physical activity, and behavioural interventions for the treatment of overweight or obesity in preschool children up to the age of 6 years. Cochrane Database Syst Rev.

[14] Wantland DJ, Portillo CJ, Holzemer WL, Slaughter R, McGhee EM (2004). The effectiveness of Web-based vs. non-Web-based interventions: a meta-analysis of behavioral change outcomes. J Med Internet Res.

[15] Lau PWC, Lau EY, Wong DP, Ransdell L (2011). A systematic review of information and communication technology-based interventions for promoting physical activity behavior change in children and adolescents. J Med Internet Res.

[16] Correa T, Hinsley AW, de Zúñiga HG (2010). Who interacts on the Web?: the intersection of users’ personality and social media use. Comput Hum Behav.

[17] Lenhart A, Purcell K, Smith A, Zickuhr K (2010). Social media & mobile internet use among teens and young adults.

[18] Moorhead SA, Hazlett DE, Harrison L, Carroll JK, Irwin A, Hoving C (2013). A new dimension of health care: systematic review of the uses, benefits, and limitations of social media for health communication. J Med Internet Res.

[19] Maher CA, Lewis LK, Ferrar K, Marshall S, De Bourdeaudhiji BI, Vandelanotte C (2014). Are health behavior change interventions that use online social networks effective? A systematic review. J Med Internet Res.

[20] Balatsoukas P, Kennedy CM, Buchan I, Powell J, Ainsworth J (2015). The role of social network technologies in online health promotion: a narrative review of theoretical and empirical factors influencing intervention effectiveness. J Med Internet Res.

[21] Boyd D, Ellison N (2007). Social network sites: definition, history, and scholarship. J Comput Mediat Commun.

[22] Eysenbach G, CONSORT-EHEALTH Group (2011). CONSORT-EHEALTH: improving and standardizing evaluation reports of Web-based and mobile health interventions. J Med Internet Res.

[23] Neumark-Sztainer D, Eisenberg ME, Fulkerson JA, Story M, Larson NI (2008). Family meals and disordered eating in adolescents: longitudinal findings from project EAT. Arch Pediatr Adolesc Med.

[24] Thompson FE, Subar AF, Smith AF, Midthune D, Radimer KL, Kahle LL, Kipnis V (2002). Fruit and vegetable assessment: performance of 2 new short instruments and a food frequency questionnaire. J Am Diet Assoc.

[25] Hunsberger M, O'Malley J, Block T, Norris JC (2015). Relative validation of Block Kids Food Screener for dietary assessment in children and adolescents. Matern Child Nutr.

[26] Neumark-Sztainer D, Wall M, Perry C, Story M (2003). Correlates of fruit and vegetable intake among adolescents. Findings from Project EAT. Prev Med.

[27] Larson NI, Neumark-Sztainer D, Hannan PJ, Story M (2007). Trends in adolescent fruit and vegetable consumption, 1999-2004: project EAT. Am J Prev Med.

[28] Blumberg SJ, Bialostosky K, Hamilton WL, Briefel RR (1999). The effectiveness of a short form of the Household Food Security Scale. Am J Public Health.

[29] Anliker J, Willis W, Montgomery S (2014). The development and testing of the behavior checklist questions for the EFNEP Evaluation/Reporting System.

[30] Bickel G, Nord M, Price C, Hamilton W, Cook J (2000). Measuring food security in the United States: guide to measuring household food security. Revised 2000.

[31] Mathews DR, Kunicki ZJ, Colby SE, Franzen-Castle L, Kattelmann KK, Olfert MD, White AA (2019). Development and testing of program evaluation instruments for the iCook 4-H curriculum. J Nutr Educ Behav.

[32] Bandura A (2004). Health promotion by social cognitive means. Health Educ Behav.

[33] Pagoto SL, Schneider KL, Oleski J, Smith B, Bauman M (2014). The adoption and spread of a core-strengthening exercise through an online social network. J Phys Act Health.

[34] Foster D, Linehan C, Kirman B, Lawson S, James G (2010). Motivating physical activity at work: using persuasive social media for competitive step counting.

[35] Boyatzis R (1998). Transforming Qualitative Information: Thematic Analysis and Code Development.

[36] Braun V, Clarke V, Cooper H, Long DL, Panter AT, Rindskopf D, Sher KJ (2012). Thematic analysis. APA Handbook of Research Methods in Psychology.

[37] Potts HWW (2011). Student experiences of creating and sharing material in online learning. Med Teach.

[38] Clifton A, Mann C (2011). Can YouTube enhance student nurse learning?. Nurse Educ Today.

[39] Livingood WC, Monticalvo D, Bernhardt JM, Wells KT, Harris T, Kee K, Hayes J, George D, Woodhouse LD (2017). Engaging adolescents through participatory and qualitative research methods to develop a digital communication intervention to reduce adolescent obesity. Health Educ Behav.

[40] Hmelo-Silver CE (2004). Problem-based learning: what and how do students learn?. Educ Psychol Rev.

[41] Karahan E, Roehrig G (2015). Constructing media artifacts in a social constructivist environment to enhance students' environmental awareness and activism. J Sci Educ Technol.

[42] Saville K (2011). Strategies for using repetition as a powerful teaching tool. Music Educat J.

[43] Law BMF, Siu AMH, Shek DTL (2012). Recognition for positive behavior as a critical youth development construct: conceptual bases and implications on youth service development. Scic World J.

[44] Mazur E, Kozarian L (2009). Self-presentation and interaction in blogs of adolescents and young emerging adults. J Adolesc Res.

[45] Rule AC, Dockstader CJ, Stewart RA (2006). Hands-onkinesthetic activities for teaching phonological awareness. Early Child Educ J.

[46] (2018). Internet/broadband fact sheet.

[47] Tsetsi E, Rains SA (2017). Smartphone internet access and use: extending the digital divide and usage gap. Mobile Media Commun.

[48] Vandelanotte C, Kirwan M, Rebar A, Alley S, Short C, Fallon L, Buzza G, Schoeppe S, Maher C, Duncan MJ (2014). Examining the use of evidence-based and social media supported tools in freely accessible physical activity intervention websites. Int J Behav Nutr Phys Act.

[49] Burrows T, Hutchesson M, Chai LK, Rollo M, Skinner G, Collins C (2015). Nutrition interventions for prevention and management of childhood obesity: what do parents want from an eHealth program?. Nutrients.

[50] Anderson M, Kumar M (2017). Digital divide persists even as lower-income Americans make gains in tech adoption.

[51] Al Mamun M, Ibrahim HM, Turin TC (2015). Social media in communicating health information: an analysis of Facebook groups related to hypertension. Prev Chronic Dis.

[52] Post SD, Taylor SC, Sanders AE, Goldfarb JM, Hunt YM, Augustson EM (2013). If you build (and moderate) it, they will come: the Smokefree Women Facebook page. J Natl Cancer Inst Monogr.

[53] Syred J, Naidoo C, Woodhall SC, Baraitser P (2014). Would you tell everyone this? Facebook conversations as health promotion interventions. J Med Internet Res.

