# Supplementary material for: Incorporating Technology Into the iCook 4-H Program, a Cooking Intervention for Adults and Children: Randomized Controlled Trial
Source: JMIR Pediatr Parent. 2019 Aug 29;2(2):e11235. doi: 10.2196/11235 (PMC6744819; doi:10.2196/11235)
Supplement: Multimedia Appendix 1 [file pediatrics_v2i2e11235_app1.pdf]

CONSORT-EHEALTH Checklist V1.6.2 Report	Manuscript Number	11235
**(based on CONSORT-EHEALTH V1.6), available at [http://tinyurl.com/consort-ehealth-v1-6].**		
**Date completed**		
7/24/2019 10:41:43		
**by**		
Lauren Moret		
Incorporating Technology into the iCook 4-H Program, Cooking Intervention for Adults and Children: Randomized Controlled Trial		
TITLE		
**1a-i) Identify the mode of delivery in the title**		
"Incorporating Technology into the iCook 4-H Program, a Cooking Intervention for Adults and Children: Randomized Controlled Trial"		
**1a-ii) Non-web-based components or important co-interventions in title**		
**1a-iii) Primary condition or target group in the title**		
"The purpose of iCook 4-H, a series of 6 cooking lessons, was to help families learn to cook, play, and eat together to assist in the prevention of childhood obesity."		
ABSTRACT		
**1b-i) Key features/functionalities/components of the intervention and comparator in the METHODS section of the ABSTRACT**		
Sections include background, objective, methods, results, and conclusion within the abstract.		
**1b-ii) Level of human involvement in the METHODS section of the ABSTRACT**		
**1b-iii) Open vs. closed, web-based (self-assessment) vs. face-to-face assessments in the METHODS section of the ABSTRACT**		
**1b-iv) RESULTS section in abstract must contain use data**		
**1b-v) CONCLUSIONS/DISCUSSION in abstract for negative trials**		
INTRODUCTION		
**2a-i) Problem and the type of system/solution**		
"Unhealthy dietary patterns in childhood are associated with less than optimal growth patterns, cognitive deUciencies, emotional unwellness, and the development of many chronic diseases [1-7]. This is of concern because few children in the United States meet all dietary intake recommendations..."		
**2a-ii) Scientific background, rationale: What is known about the (type of) system**		
"Social media technology is a tool that can be used in health promotion interventions for children because children are often one of the earliest adopters of technology [16-18]. Although there is limited research on health-related interventions using social media..."		

**Does your paper address CONSORT subitem 2b?**		
"The purpose of this study was to describe the incorporation of technology, including uses of and barriers to the use of technology, during an intervention by program participants..."		
METHODS		
**3a) CONSORT: Description of trial design (such as parallel, factorial) including allocation ratio**		
"....Although this study was not prospectively registered as a randomized controlled trial, the institutional review boards at all participating universities approved the study procedures. All participants provided assent and consent to participate."		
**3b) CONSORT: Important changes to methods after trial commencement (such as eligibility criteria), with reasons**		
"The iCook 4-H intervention program was a pre-post, follow-up intervention study conducted over 2 years for dyads (9- to 10-year-old children and their primary adult meal preparer) across 5 states in the United States (Maine, Nebraska, South Dakota, Tennessee, and West Virginia)..."		
**3b-i) Bug fixes, Downtimes, Content Changes**		
**4a) CONSORT: Eligibility criteria for participants**		
"Participants recruited for this study (1) were free from life-threatening illness or conditions, (2) were free from food allergies or activity-related medical restrictions that would prevent participation in a face-to-face nutrition and Utness program, (3) were willing to eat meat and dairy foods, and (4) had regular access to a computer with an internet connection."		
**4a-i) Computer / Internet literacy**		
**4a-ii) Open vs. closed, web-based vs. face-to-face assessments:**		
"We developed a password-protected website for participants to use to reinforce session content and increase connections between participants across the 5 states through status updates and comments...."		
**4a-iii) Information giving during recruitment**		
**4b) CONSORT: Settings and locations where the data were collected**		
"All participants completed baseline (0-month), postintervention (4-month), and follow-up (12- and 24-month) assessments. Assessments at these time points included measuring childrens' height, weight, waist circumference, and blood pressure as well as completing surveys. Survey questions assessed demographics, dietary intake, food security, cooking frequency, and cooking selfejcacy..."		
**4b-i) Report if outcomes were (self-)assessed through online questionnaires**		
"Participants were asked to post videos, recipes, status updates about personal goals, and reactions to other participants' postings between sessions. Videos were to be 3 to 5 minutes in length and reuect topics learned in the sessions. Video cameras were provided..."		
**4b-ii) Report how institutional affiliations are displayed**		
**5) CONSORT: Describe the interventions for each group with sufficient details to allow replication, including how and when they were actually administered**		
**5-i) Mention names, credential, affiliations of the developers, sponsors, and owners**		

**5-ii) Describe the history/development process**		
**5-iii) Revisions and updating**		
**5-iv) Quality assurance methods**		
**5-v) Ensure replicability by publishing the source code, and/or providing screenshots/screen-capture video, and/or providing flowcharts of the algorithms used**		
**5-vi) Digital preservation**		
**5-vii) Access**		
"Beginning 1 month after the 12-week program concluded and continuing for 18 months, participants received an additional 21 months of website activities, monthly newsletters, and quarterly in-person booster sessions..."		
**5-viii) Mode of delivery, features/functionalities/components of the intervention and comparator, and the theoretical framework**		
"Participants were asked to post videos, recipes, status updates about personal goals, and reactions to other participants' postings between sessions..." Researchers compared differences between the two groups' videos.		
**5-ix) Describe use parameters**		
**5-x) Clarify the level of human involvement**		
**5-xi) Report any prompts/reminders used**		
"Treatment dyads participated in six 2-hour sessions held every other week, over a period of 12 weeks...."		
**5-xii) Describe any co-interventions (incl. training/support)**		
"The Extension personnel were community nutrition educators or para-professionals from the participating landgrant institutions. At the end of each session, leaders and dyads completed online process surveys, which included open-ended feedback questions on technology. Leaders also participated in monthly phone calls with researchers for process evaluation..."		
**6a) CONSORT: Completely defined pre-specified primary and secondary outcome measures, including how and when they were assessed**		
"Although all treatment group children were asked to access the website and submit postings of their videos, only 69.0% (71/103) went on the iCook website. Of those who did post videos, 59% (42/71) posted 1 to 3 videos, 24% (17/71) posted 4 to 7 videos, and 17% (12/71) posted 8 or more videos..."		
**6a-i) Online questionnaires: describe if they were validated for online use and apply CHERRIES items to describe how the questionnaires were designed/deployed**		
**6a-ii) Describe whether and how "use" (including intensity of use/dosage) was defined/measured/monitored**		
**6a-iii) Describe whether, how, and when qualitative feedback from participants was obtained**		
**6b) CONSORT: Any changes to trial outcomes after the trial commenced, with reasons**		

"All participants completed baseline (0-month), postintervention (4-month), and follow-up (12- and 24-month) assessments. Assessments at these time points included measuring childrens' height, weight, waist circumference, and blood pressure as well as completing surveys. Survey questions assessed demographics, dietary intake, food security, cooking frequency, and cooking selfejcacy..."		
**7a) CONSORT: How sample size was determined**		
**7a-i) Describe whether and how expected attrition was taken into account when calculating the sample size**		
**7b) CONSORT: When applicable, explanation of any interim analyses and stopping guidelines**		
"Although all treatment group children were asked to access the website and submit postings of their videos, only 69.0% (71/103) went on the iCook website. Of those who did post videos, 59% (42/71) posted 1 to 3 videos, 24% (17/71) posted 4 to 7 videos, and 17% (12/71) posted 8 or more videos..."		
**8a) CONSORT: Method used to generate the random allocation sequence**		
"Although this study was not prospectively registered as a randomized controlled trial, the institutional review boards at all participating universities approved the study procedures. All participants provided assent and consent to participate."		
**8b) CONSORT: Type of randomisation; details of any restriction (such as blocking and block size)**		
"We randomly assigned those who met the inclusion criteria to the control group (n=77) or intervention group (n=151), using a pattern of 1 control for every 2 treatment dyads."		
**9) CONSORT: Mechanism used to implement the random allocation sequence (such as sequentially numbered containers), describing any steps taken to conceal the sequence until interventions were assigned**		
The paper does not address this question.		
**10) CONSORT: Who generated the random allocation sequence, who enrolled participants, and who assigned participants to interventions**		
The primary investigator, Dr. Sarah Colby.		
**11a) CONSORT: Blinding - If done, who was blinded after assignment to interventions (for example, participants, care providers, those assessing outcomes) and how**		
**11a-i) Specify who was blinded, and who wasn't**		
Dyads were assigned randomly		
**11a-ii) Discuss e.g., whether participants knew which intervention was the "intervention of interest" and which one was the "comparator"**		
**11b) CONSORT: If relevant, description of the similarity of interventions**		
Not relevant		
**12a) CONSORT: Statistical methods used to compare groups for primary and secondary outcomes**		
"We calculated frequency statistics for demographics, technological variables, and website usage and preferences for children and adults in the treatment group...."		
**12a-i) Imputation techniques to deal with attrition / missing values**		
We did not address this in the paper. We did write: "Although all treatment group children were asked to access the website and submit postings of their videos, only 69.0% (71/103) went on the iCook website. Of those who did post videos, 59% (42/71) posted 1 to 3 videos, 24% (17/71) posted 4 to 7 videos, and 17% (12/71) posted 8 or more videos. One person posted 26 videos and 1 person posted 29 videos."		

**12b) CONSORT: Methods for additional analyses, such as subgroup analyses and adjusted analyses**		
We did not address this in the paper.		
RESULTS		
**13a) CONSORT: For each group, the numbers of participants who were randomly assigned, received intended treatment, and were analysed for the primary outcome**		
We did not address this in the paper.		
**13b) CONSORT: For each group, losses and exclusions after randomisation, together with reasons**		
We did not address this in the paper.		
**13b-i) Attrition diagram**		
**14a) CONSORT: Dates defining the periods of recruitment and follow-up**		
We provide information about the study lasting over a 24-month period, but we do not provide exact dates.		
**14a-i) Indicate if critical "secular events" fell into the study period**		
**14b) CONSORT: Why the trial ended or was stopped (early)**		
The design of the trial was 24-months. We ended the trial after this time.		
**15) CONSORT: A table showing baseline demographic and clinical characteristics for each group**		
Yes, we provide a table for the adult participants showing sex, ethnicity, marital status, educational level, and household food security information. We do not provide a table for the child participants.		
**15-i) Report demographics associated with digital divide issues**		
We do not report demographics related to the digital divide. But some Undings indicate adults and children have similar barriers to participating in technology.		
**16a) CONSORT: For each group, number of participants (denominator) included in each analysis and whether the analysis was by original assigned groups**		
**16-i) Report multiple "denominators" and provide definitions**		
I do not know how to respond to this inquiry.		
**16-ii) Primary analysis should be intent-to-treat**		
**17a) CONSORT: For each primary and secondary outcome, results for each group, and the estimated effect size and its precision (such as 95% confidence interval)**		
No, our paper does not address this.		
**17a-i) Presentation of process outcomes such as metrics of use and intensity of use**		
**17b) CONSORT: For binary outcomes, presentation of both absolute and relative effect sizes is recommended**		
No, our paper does not address this.		
**18) CONSORT: Results of any other analyses performed, including subgroup analyses and adjusted analyses, distinguishing pre-specified from exploratory**		
We provide frequency information and chi square analysis.		
**18-i) Subgroup analysis of comparing only users**		
**19) CONSORT: All important harms or unintended effects in each group**		

No, our paper does not address this. We fall on our IRB consent form document to support any harmfulness that may come as a result of participating in this research.		
**19-i) Include privacy breaches, technical problems**		
**19-ii) Include qualitative feedback from participants or observations from staff/researchers**		
DISCUSSION		
**20) CONSORT: Trial limitations, addressing sources of potential bias, imprecision, multiplicity of analyses**		
**20-i) Typical limitations in ehealth trials**		
No, our paper does not address this. I think this content could be argued for eternity.		
**21) CONSORT: Generalisability (external validity, applicability) of the trial findings**		
**21-i) Generalizability to other populations**		
**21-ii) Discuss if there were elements in the RCT that would be different in a routine application setting**		
**22) CONSORT: Interpretation consistent with results, balancing benefits and harms, and considering other relevant evidence**		
**22-i) Restate study questions and summarize the answers suggested by the data, starting with primary outcomes and process outcomes (use)**		
We believe we have enacted beneUcence throughout this process.		
**22-ii) Highlight unanswered new questions, suggest future research**		
Other information		
**23) CONSORT: Registration number and name of trial registry**		
I am not certain of how to respond to this inquiry. I am unaware of a trial registry number; I only know our IRB approval number.		
**24) CONSORT: Where the full trial protocol can be accessed, if available**		
See Dr. Colby's work for more information.		
**25) CONSORT: Sources of funding and other support (such as supply of drugs), role of funders**		
USDA Grant		
**X26-i) Comment on ethics committee approval**		
**x26-ii) Outline informed consent procedures**		
**X26-iii) Safety and security procedures**		
**X27-i) State the relation of the study team towards the system being evaluated**		
